# [Corrigendum] FKBP11 protects intestinal epithelial cells against inflammation‑induced apoptosis via the JNK‑caspase pathway in Crohn's disease

**DOI:** 10.3892/mmr.2024.13172

**Published:** 2024-01-26

**Authors:** Xiaotong Wang, Xiaopeng Cui, Chuanwu Zhu, Ming Li, Juan Zhao, Zhongyi Shen, Xiaohang Shan, Liang Wang, Han Wu, Yanting Shen, You Ni, Dongmei Zhang, Guoxiong Zhou

Mol Med Rep 18: 4428–4438, 2018; DOI: 10.3892/mmr.2018.9485

Subsequently to the publication of the above article, the authors realized that Fig. 4 in their paper had been assembled containing two erroneously placed gel slices; essentially, the GAPDH bands featured in Fig. 4A had also been included in Fig. 5, and the data for the FKBP11 bands in Fig. 4A had also been included to show the GRP78 bands in Fig. 4.

The authors were able to revisit their original data and to correct the data that had been featured incorrectly in Fig. 4. The corrected version of Fig. 4, now showing the true data for the GRP78 protein bands in Fig. 4C and the correct GAPDH protein bands for Fig. 4A, is shown on the next page. Note that these errors did not significantly affect the results or the conclusions reported in this paper. All the authors agree to the publication of this Corrigendum, and are grateful to the Editor of *Molecular Medicine Reports* for allowing them the opportunity to correct this error. Moreover, the authors apologize to the readership for any inconvenience caused.

## Figures and Tables

**Figure 4.**
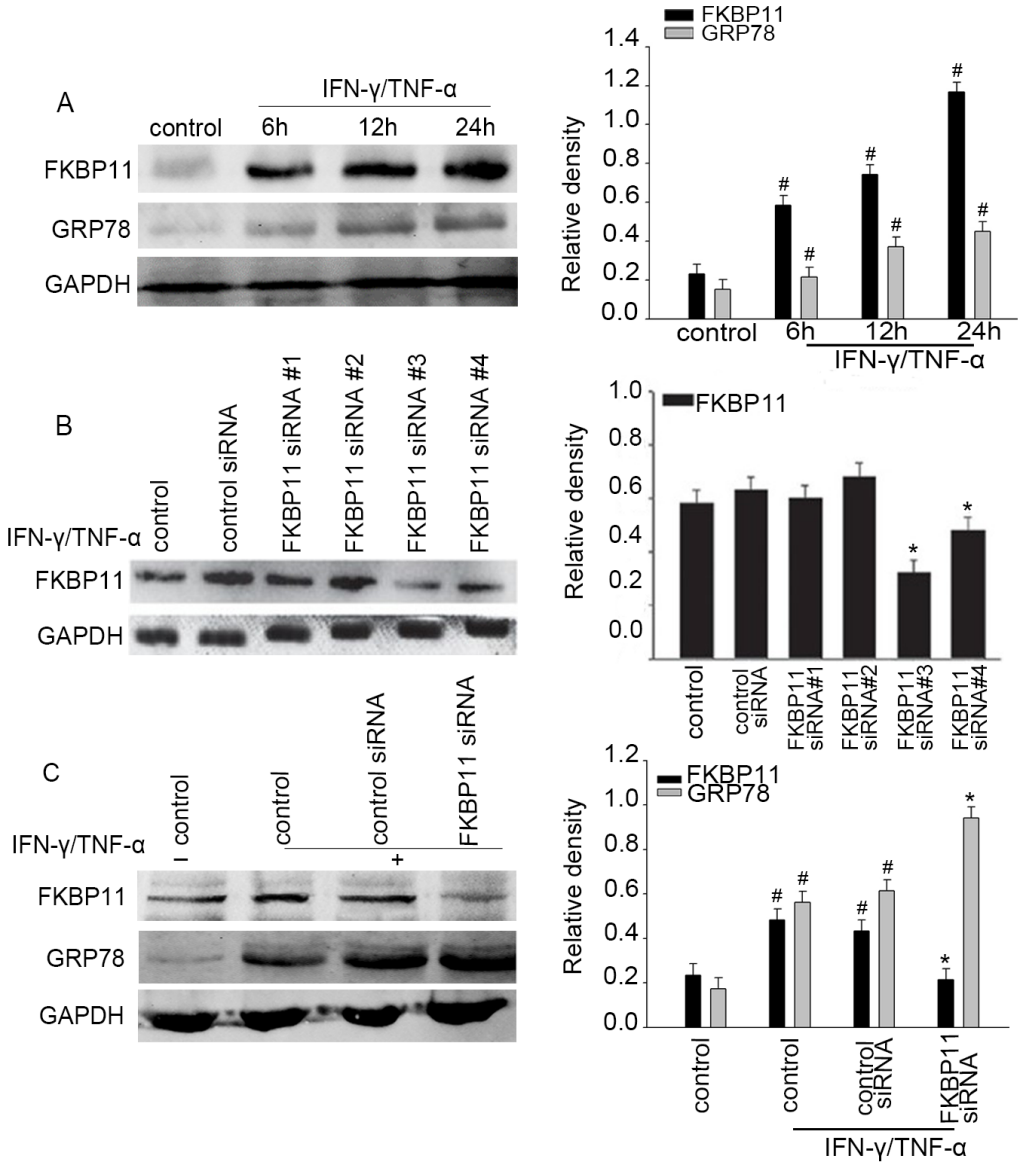
Association of FKBP11 expression levels with ER stress in IFN-γ/TNF-α-treated HT-29 cells. (A) HT-29 cells were treated with IFN-γ (2.5 ng/ml) and TNF-α (50 ng/ml) for different time intervals (0, 6, 12 and 24 h) to construct a cell model of ER stress. Western blot analyses revealed a significant upregulation of FKBP11 and GRP78 protein levels in IFN-γ/TNF-α-treated cells in a time-dependent manner. (B) FKBP11 expression following transfection with FKBP11 siRNA in HT-29 cells was revealed by western blot analyses, and transfection with FKBP11 siRNA#3 was revealed to exhibit the most significant downregulation of FKBP11 expression. (C) HT-29 cells were transfected with control siRNA or FKBP11 siRNA for 48 h, and then treated with IFN-γ/TNF-α for 24 h. Western blot analyses revealed that FKBP11 and GRP78 expression levels increased following IFN-γ/TNF-α treated cells compared with the untreated control, and GRP78 expression was significantly increased following transfection of FKBP11 siRNA compared with control siRNA. Bar graphs reveal the densities of FKBP11 or GRP78 protein levels vs. GAPDH. Data are presented as mean ± standard error (n=3). *P<0.05 vs. control siRNA; ^#^P<0.05 vs. IFN-γ/TNF-α untreated control. IFN-γ, interferon-γ; TNF-α, tumor necrosis factor-α; FKBP11, FK506 binding protein 11; GRP78, 78 kDa glucose-regulated protein; siRNA, small interfering RNA.

